# Using nonlinear methods to quantify changes in infant limb movements and vocalizations

**DOI:** 10.3389/fpsyg.2014.00771

**Published:** 2014-08-12

**Authors:** Drew H. Abney, Anne S. Warlaumont, Anna Haussman, Jessica M. Ross, Sebastian Wallot

**Affiliations:** ^1^Cognitive and Information Sciences, University of California, MercedMerced, CA, USA; ^2^Independent ScholarAarhus, Denmark; ^3^Department of Culture and Society, Interacting Minds Center, Aarhus UniversityAarhus, Denmark

**Keywords:** motor development, infant vocalization, nonlinear methods, recurrence, Allan factor

## Abstract

The pairing of dynamical systems theory and complexity science brings novel concepts and methods to the study of infant motor development. Accordingly, this longitudinal case study presents a new approach to characterizing the dynamics of infant limb and vocalization behaviors. A single infant's vocalizations and limb movements were recorded from 51-days to 305-days of age. On each recording day, accelerometers were placed on all four of the infant's limbs and an audio recorder was worn on the child's chest. Using nonlinear time series analysis methods, such as recurrence quantification analysis and Allan factor, we quantified changes in the stability and multiscale properties of the infant's behaviors across age as well as how these dynamics relate across modalities and effectors. We observed that particular changes in these dynamics preceded or coincided with the onset of various developmental milestones. For example, the largest changes in vocalization dynamics preceded the onset of canonical babbling. The results show that nonlinear analyses can help to understand the functional co-development of different aspects of infant behavior.

## Introduction

The human infant is a developing complex system that moves, perceives, explores, and interacts with its environment. By the end of the first year, an infant has experienced 31,536,000 s of the dynamics and structure of its world. During this time, we see a rich, multidimensional developmental trajectory, including changes in the physical body and improved motor control (Goldfield, 1995; Thelen, 1995; Adolph and Berger, 2011) and changes to perceived and created acoustic structures corresponding to increasingly sophisticated communication with others (Vihman, 1996; Oller, 2000).

Developmental psychology—perhaps more than any other branch of psychology—has had to grapple with the dynamic nature of human behavior and cognition. Qualitative changes occur during the lifespan of an individual, and co-development of different systems (e.g., motor, language, social, cognitive) is the rule, rather than the exception (Iverson, 2010; Parladé and Iverson, 2011; Frank et al., 2013; Walle and Campos, 2013). Each system is constantly changing as various interrelated skills develop and regularities emerge (Kugler and Turvey, 1987; Smith and Thelen, 2003, speak of *soft assemblies*).

This article reports a case study that uses a novel combination of nonlinear time series analysis methods to analyze patterns of change in infant limb behavior and vocalizations as a function of age. A multi-modal longitudinal corpus of time series was collected from one infant, SW, from 2 months of age to the end of the first year. In addition, SW's parents^1^ documented a variety of motor and language milestones exhibited throughout the data collection period (as defined in Adolph et al., 2008; Buder et al., 2013). Considering the properties of this corpus, we highlight: (1) the changing stabilities and multiscale properties of limb and vocalization behaviors across development, (2) the relationships across these two modalities, and (3) the relationship between the changing vocal and limb dynamics and parent-reported developmental milestones.

Before explaining in more detail the corpus and the analysis techniques, we start by providing an introduction to variability in developmental science and an overview of developmental milestones and modalities that are relevant to the findings in the present case study.

### Infant development characterized by variability

In the current paper, we adopt a dynamical systems theory (DST) approach and tools from complexity science to characterize infant limb and vocal development. One feature that distinguishes the DST approach from other views of motor development is how variability across development is viewed (for review, see Vereijken, 2010; Stergiou et al., 2013). Many past theories of motor development ascribed the observation of more variability or greater deviation from the normative sequence of developmental achievements as indicative of developmental dysfunction (Shirley, 1931; McGraw, 1945). Most relevant to the current paper is that these approaches view variability in a developing infant as an indicator of non-standard development.

It is not always the case that an infant will achieve a particular motor milestone in the normative age range, and the sequence of such milestones can vary, too (Vereijken and Adolph, 1999). Although there are common sequences of milestones that occur roughly within similar age ranges, the amount of intra-individual and inter-individual variability observed across development points to a departure from stage theoretic frameworks and more toward frameworks that characterize change in a different way. The process of change over development might be usefully characterized by measures that account for the changes in variability of the motor system (Vereijken, 2010; Stergiou et al., 2013).

A DST approach to motor development emphasizes the process of change, or rather the *dynamics* of change (Thelen et al., 1987; Smith and Thelen, 1993). The sources of order and stability in motor behaviors over temporal and spatial scales are emphasized (Bassingthwaighte et al., 1994). Instead of ascribing external behaviors primarily to internal motor programs (Schmidt, 1975), the emphasis is on the ensemble properties of movement (Bernstein, 1967) where muscles and effectors are integrated into coordinative structures (Kelso and Tuller, 1984; Saltzman and Kelso, 1987).

Characterizing development of biological systems using variability affords a detailed look at the dynamics of change. Variability can be assessed using both linear and nonlinear measures. Linear measures of variability such as standard deviation and coefficient of variation report the deviations from a central point such as the mean. However, assumptions such as the independence of measurement lead to the notion that variations are random. This is problematic because it is known that variations are typically non-random and have important characteristic structures (Van Orden et al., 2003, 2005; Delignières and Torre, 2009). Nonlinear measures of variability take into account the temporal structure and the distributional organization of variability. Some examples of nonlinear measures of variability are fractal geometric methods, entropy estimation, and recurrence quantification analysis. Using either linear or nonlinear measures to identify changes in variability that relate to changes in the state of a complex system is a hallmark of DST. DST has specific expectations for when changes in a system will occur (Haken et al., 1985) and has been useful in characterizing behavioral and cognitive development (e.g., Thelen et al., 1993; Vereijken and Thelen, 1997; Harbourne and Stergiou, 2003; Stephen et al., 2009, 2012; Parladé and Iverson, 2011).

In this case study, we investigate dynamic patterns of limb movements and prelinguistic vocalizations not only as independent components but also as interdependent systems that develop together throughout infancy. Note that while the terms “limb” and “vocal” are used in this paper to distinguish two general classes of behaviors, we consider these two systems as components of various coordinated structures in a developing complex system.

The utilization of nonlinear methods to study infant motor development is not new and has already had a profound impact on the field. However, *nonlinear methods* for measuring variability, popular in dynamical systems approaches in a variety of other fields, have so far been under-utilized in studying vocal and limb co-development. The current case study provides initial insights into the ways in which applying nonlinear methods to study patterns of variability across various motor systems might provide interesting and novel information about developmental achievements.

We will now provide a brief description of the notable patterns of behavior observed in various motor and vocal milestones throughout the first year of life. We do not aim to provide a comprehensive review of all documented milestones, but rather we focus on milestones that are relevant to the observations reported in the current paper. We also focus on limb and vocal development together in order to engage in an existing dialog within the literature that suggests that the two systems are coupled and that this coupling of limb and vocal behaviors facilitates language development (Iverson and Thelen, 1999; Iverson, 2010; Walle and Campos, 2013).

### Limb movement development and behaviors

During the first year of a human's life, patterns of limb behavior change dramatically. Newborns display reflexive, spontaneous movements before gross motor movement patterns are observable (Iverson and Thelen, 1999). Manual behaviors become pronounced, and rhythmic patterns of various types emerge and progress (Thelen, 1979; Thelen and Fisher, 1983). With increasing age, complex, coordinative patterns of motor behavior across effectors and in coordination with the infant's environment are observed (Lewkowicz, 2000; Adolph et al., 2012). In this case study, motor milestones such as rolling, reaching, sitting, and crawling were documented.

Frequency of rhythmic and spontaneous limb movement increases in the first few months of infancy (Thelen, 1979) followed by more coordinated patterns of inter-limb movements (Piek and Gasson, 1999; Piek et al., 2002; Kanemaru et al., 2012). For example, Kanemaru et al. (2012) observed that by three-months of age, a dissociation of the upper and lower limbs occurs, as evidenced by increasing correlations between the velocities of the two arms and between the two legs. Such dissociations have been shown to facilitate intentional functions such a playing with a toy or manipulating an object (Watanabe and Taga, 2009). The coordinated symmetry of inter-limb movement patterns provides an example of how the degrees of freedom of the limb effectors are constrained (Bernstein, 1967), facilitating coordinated action.

Another example relevant to this case study is unsupported sitting. Unsupported sitting or independent sitting is first achieved by most infants at around six- to seven-months of age (Bayley, 1993). Postural control is a prerequisite behavior that facilitates unsupported sitting. In other words, unsupported sitting is nested, or embedded, in postural control (Bernstein, 1967; Gibson and Pick, 2000). Therefore, the nested action of unsupported sitting affords many new movements and intentional actions (Reed, 1982, 1996). For example, stable reaching (Spencer et al., 2000) and visual exploration (Bertenthal and Von Hofsten, 1998) are associated with a progression of stable patterns of unsupported sitting. The variability of center of pressure (COP) observed throughout the development of unsupported sitting has been analyzed using nonlinear methods, and suggests that COP fluctuations are non-random and move from flexible, adaptive movements to more stable, regular patterns of behavior (Harbourne and Stergiou, 2003). These developmental patterns of variability suggest a dynamic interplay between flexibility and stability of movements in order to achieve new behaviors and adapt to the environment. Unsupported sitting might also facilitate language development (Iverson, 2010) by allowing deeper breathing from increased lung capacity and more controlled respiration (Boliek et al., 1996), which can enhance utterance productions and other vocalization properties (Yingling, 1981).

### Vocalization development and behaviors

As with other motor development, prespeech vocal development has also been described as progressing through specific phases. These phases, similar to other motor behaviors, are nested and require the acquisition and control of earlier stages before achieving more complex behaviors. Oller (2000) summarized four stages of vocal development identified by various investigators (e.g., Oller, 1980; Stark, 1980; Elbers, 1982): (1) Phonation stage, (2) Primitive articulation stage, (3) Expansion stage, and (4) Canonical stage. The phonation stage occurs from birth to around 2 months of age, and is marked by primitive protophones (precursors to speech sounds) such as quasivowels and glottal stops. The primitive articulation stage occurs around two- to three-months of age. During this stage, articulation during vocalization emerges and sounds such as “gooing” are observed. The expansion stage occurs around three- to eight-months of age, and is marked by the onset of pitch, amplitude, and voice quality contrasts, e.g., squeals, growls, and yells. The canonical stage begins at around seven months of age for typically developing infants, and is marked by the well-formed production of syllables containing both consonant and vowel sounds, such as “dada” or “baba.”

The onset of canonical babbling is perhaps the most striking and best-studied prelinguistic vocal milestone. Its development has been argued to be quite robust since factors such as low socioeconomic status, mild hearing impairments, and exposure to different language environments have not been found to affect the age of onset of canonical babbling (Oller, 2000). On the other hand, severe or profound hearing impairment does significantly delay the onset of canonical babbling, suggesting a relationship between vocal motor behavior and sensation (Oller and Eilers, 1988), and canonical babbling has also been shown to be delayed in children who are later diagnosed with autism (Patten et al., 2014) and produced at lower rates in children who go on to develop a reading disorder (Smith et al., 2010). It has been shown that the specific vowels and consonants that make up infants' syllables during the canonical stage are the same ones that tend to make up their first words (Vihman et al., 1985).

As with the motor milestones discussed earlier, the variability in infant vocalizations is striking. For example, there is quite a bit of variability in the age at which infants enter the canonical stage (Oller et al., 1994). There is also variability both across individuals and within an individual recorded at different points in time in rate of vocalization (volubility), in representation of different vocal types, and in the physiological control underlying vocalizations (Locke, 1989; Boliek et al., 1996; Oller et al., 2013; Franklin et al., 2014). It is also worth noting that as with many of the motor milestones discussed earlier, the prevalence of canonical babbling behavior, quantified as the canonical babbling ratio, increases steadily over the second half of the first year (Oller et al., 1997). Considering the many reports documenting the variability of infant vocalizations, it seems worthwhile to try applying nonlinear methods for studying patterns of temporal variability to the study of human vocal development.

### Relationship between limb and vocalization behaviors

There appears to be a cross-modal relationship between vocal and limb behaviors. It has been repeatedly shown that there is indeed coordination between the development of rhythmic limb and vocal productions, with rhythmic arm movement and multisyllabic canonical babbling emerging at similar ages (Cobo-Lewis et al., 1996; Iverson and Thelen, 1999; Ejiri and Masataka, 2001; Iverson and Fagan, 2004). It has also been shown that a variety of other motor milestones and language acquisition milestones are related to each other (Iverson and Goldin-Meadow, 2005; Iverson, 2010; Walle and Campos, 2013).

Iverson and Thelen (1999) proposed a conceptual model wherein coordinated limb and vocal development in infancy evolves to what is later observed as speech-gesture coupling in adults (McNeill, 1992). Inspired by coupled oscillators and self-organization (Kugler and Turvey, 1987; Kelso, 1995), their model proposes four phases that differ in the amount of flexibility, co-activation, and coupling between the limb and vocal effectors, culminating in the tightly coupled behaviors of gestures and speech exhibited by adults. Previous research suggests that the emerging co-activation of the limb and vocal systems seems to play a role in language development.

Other than the co-activation of limb and vocal effectors that might give rise to more robust communicative skills, there are other ways that the limb movements and vocalizations might interact with each other. The achievement of motor milestones, whether abrupt or gradual, can lead to specific affordances for the vocal system. For example, postural development seen in unsupported sitting can facilitate skills associated with more complex vocalization productions such as increased syllable production per breath and greater control of utterance production (Yingling, 1981). In some cases, therefore, new possibilities in the vocal system may be nested within other motor milestones; the skill of unsupported sitting or any other action capability may have important consequences for future actions across all systems (Reed, 1982; Turvey, 1992; Gibson and Pick, 2000; see e.g., Nickel et al., 2013; Atun-Einy et al., 2014; Koterba et al., 2014). Therefore, in the current study, we consider how specific motor and language milestones might have “cascading effects” (Koterba et al., 2014) on the development of other skilled and controlled behaviors.

We focus on these two components—limb movements and vocalizations—for a number of reasons. First, much research has focused on the development of the motor-vocal system as the development of the coordination of speech articulators (Kelso et al., 1984, 1986; Saltzman and Munhall, 1989), however, less work has focused on how the coordination of motor effectors across the entire body (including limb and vocal motor effectors) might facilitate the development of various limb and vocalization skills. Second, the cascading effects of other motor milestones, such as transitioning from crawling to walking, has been shown to affect cognitive, social, and linguistic skills (Biringen et al., 1995; Walle and Campos, 2013; Kretch et al., 2014). The study of limb and vocal motor dynamics and developmental milestones might provide insight into nested actions across the entire system that have otherwise not been observed which can lead to a more robust understanding of the coordinative structures that facilitate important developmental achievements. Finally, to our knowledge, no work has used tools from complexity science to focus on the proximate relationships between limb and vocal motor behaviors and how these dynamics might relate to important developmental milestones. There are many motor systems for which it would be interesting to study both within-modality dynamics and cross-modal relationships. Given the existing research suggesting a relationship between limb movements and vocalizations and the existence of tools that make collecting daylong naturalistic recordings of both limb movements and vocalizations feasible, these two behavioral modalities seemed like a good starting point.

In studying the development of such inherently interdependent behaviors, conceptual and analytical tools developed for the study of other complex systems are bound to offer additional insights (Carello and Moreno, 2005). In the following sections, we briefly summarize how principles borrowed from the framework of complex systems theory can be applied to the understanding of development, and how these principles can be measured in infant movements and vocalizations.

### Complexity science and embodied cognition

Complexity science is an umbrella term that encompasses many inter-related disciplines and subsequent methodologies such as dynamical systems theory, nonlinear dynamics, and statistical mechanics. The methods and theoretical frameworks from the complexity sciences have proven useful in studying child development (Smith and Thelen, 2003; Vereijken, 2010; DiDonato et al., 2013; Stergiou et al., 2013) as well as in studying adult behavior (Kugler et al., 1980). Although dynamical systems theory has been fruitfully applied to developmental psychology for many years now (Thelen, 1995) and considerable work in the developmental sciences has made great strides in understanding the appropriate sampling properties of time and data series (Adolph et al., 2008), less work has focused on applying the analytic tools from complexity science to study infant limb and vocal behavior and the interplay between them. The following section describes two analytic methods that will inform us about the stability/instability and history-dependence of SW's behaviors: recurrence quantification analysis and Allan factor analysis, respectively.

#### Recurrence quantification analysis

Recurrence quantification analysis (RQA) is a nonlinear analysis that provides information about the repetition of patterns in a system recorded over time (Zbilut et al., 1998). Put simply, a system that tends to follow the same dynamical pattern over time will produce more recurrences, whereas a system with a highly variable dynamical pattern will produce fewer recurrences. By quantifying the recurrence of a system's behavior, a variety of measures indexing the structure, complexity, and stability can be computed.

**Figure 1:**
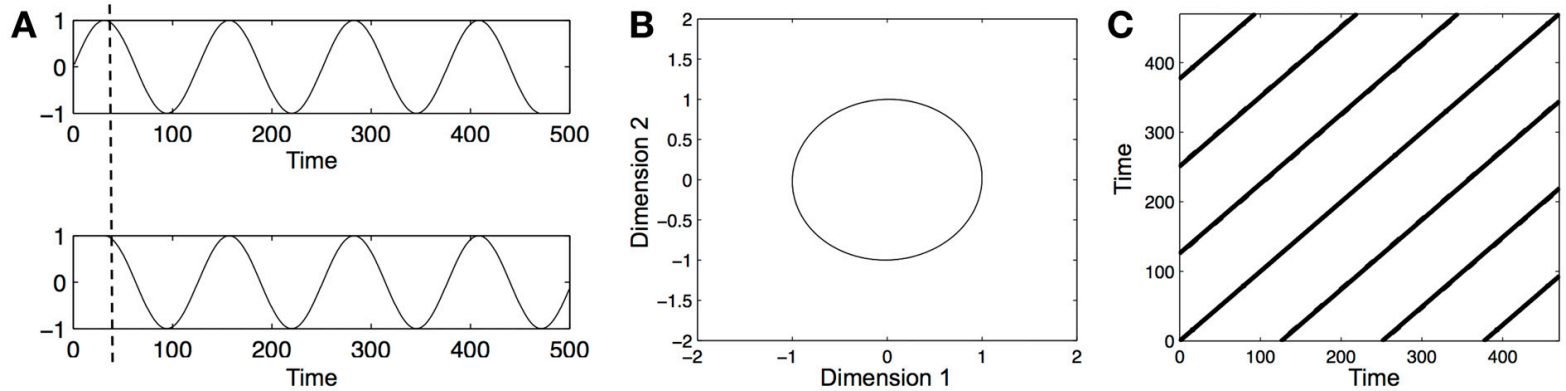
**Illustration of time series embedding, phase-space reconstruction, and recurrence plot analysis. (A)** A sine wave over several periods (top panel) and a delayed copy of that time series. The original time series and its time-delayed copy are plotted against each other to yield a 2-dimensional phase-space. **(B)** Phase-space portrait of a sine-wave. The circular shape of the profile shows that the sine-wave is highly stable and repetitive, repeating itself perfectly along a single circular path. Please note that the labeling of the dimensions as 1 and 2 is arbitrary. **(C)** Recurrence plot (RP) of the phase-space portrait. In a RP, time at lag0 runs along the central diagonal. The presence of the diagonal line states the simple fact that a time series is always the same with itself at lag0. The striped pattern that repeats itself off the diagonal toward the upper left and the lower right indicates that the time series is perfectly repeating itself, and the distance between the stripes (i.e., the white spaces between them) indicates the lag at which the time series repeats itself, and is equal to the period of the sine-wave. Since all recurrent points fall onto diagonally adjacent lines, the %DET values = 99.9% (as the sine-wave is perfectly deterministic, the values should be 100%, but spurious individual recurrence points can appear on the edges of the RP, leading to the negligible deviation from the expected value).

However, before these measures can be obtained, we need to reconstruct the phase-space of each one-dimensional time series (e.g., the time series of leg acceleration of an infant). A phase-space is a multidimensional representation of the dynamics of a system. The multi-dimensional phase-space of a one-dimensional time series can be reconstructed by the method of time-delayed embedding (Takens, 1981). Practically, this means that the original one-dimensional time series is plotted against itself several times at a fixed lag. Hence, as a first step, an appropriate dimensionality (the dimension-parameter) and an appropriate lag (the delay-parameter) need to be estimated. This can be done using the average mutual-information function (to estimate the delay parameter) and the false-nearest neighbor function (to estimate the dimension parameter), where, as a rule of thumb, the first local minimum of each function corresponds to the appropriate delay and dimension of the time series, respectively. For example, say we have a one-dimensional time series *x* of 100 data points that should be embedded with delay = 10 and dimension = 3. To reconstruct the 3-dimensional phase space, the original series is plotted against itself two more times, each time shifted by a delay of 10 (i.e., *x*_1–80_ vs. *x*_11–90_ vs. *x*_21–100_). Figure 1A illustrates the delaying of a time series, and Figure 1B shows the resulting phase-space.

In a second step, the multi-dimensional phase-space is converted into a 2-dimensional representation, the recurrence plot (RP), which is then ultimately used to derive statistics about the temporal patterns in the time series. RPs visualize how the time series “moves” through the phase space. In particular, RPs represent how patterns in a time series repeat themselves in phase-space. As real-world data is never perfectly repetitive—due to intrinsic fluctuations of the system that produced the data and due to measurement noise—one has to define a threshold for what activity is to be considered as recurrent in a time series. This threshold is the radius parameter and corresponds to a distance in the phase-space. All points in the phase-space that are farther away than that distance are counted as being different, and all points that are closer to each other than that distance are counted as the same; the latter are called recurrence points. Recurrence points are thus the core of RQA from which all other statistics are derived. The most basic measure of recurrence of a time series is its percentage of recurrent points (%REC), i.e., all distances in phase-space that are shorter than the radius parameter divided by the sum of distances in phase-space.

RQA yields many output variables, such as %REC. However, in the current study, we were specifically interested in how much infant vocalization and arm movements are structured in terms of larger, systematics patterns of limb and vocal activity. Hence, we used the measure of percent determinism (%DET), which is the sum of all recurrence points that form diagonally adjacent lines in the recurrence plot divided by the sum of all recurrent points. %DET is thus a measure of the sequential structuredness of behavior, i.e., how “lawfully” movements and vocalizations evolve in time. A behavior with high %DET is interpreted as being highly stable. Figure 1C shows an example recurrence plot with high %DET.

RQA can also yield other measures of temporal patterning. In this study, however, we will focus on the %DET measure for the following reasons: First, considering RQA is only one analysis used in this study, we chose to only compute one measure, %DET, for the sake of a concise and clear presentation. Second, it has been shown for the case of infant motor behavior that RQA measures of stability are redundant, and lead to the same results (see Assmann et al., 2007). Third—and most importantly—some of the other RQA measures (such as Maxline) are dependent on the length of the time series, and the lengths of our recordings vary by several hours. In contrast to that, %DET is calculated as a percentage, and is thus independent of the length of a recording, given that a certain minimum of data points has been collected.

From a developmental point of view, we would expect a high amount of instability to correspond to more exploratory behavior surrounding a transition in behavior, while high amounts of stability might indicate that behavior has settled into patterns of structured, skilled behavior (Thelen, 1995). For further information on RQA, we direct the interested reader to Webber and Zbilut (2005) for a comprehensive introduction and mathematically thorough treatment of RQA.

#### Allan factor analysis

Allan factor analysis (AF) estimates the scaling of event clustering across multiple temporal scales. As a sub-category of fractal analysis, it affords the ability to estimate fractal exponents of behaviors of interest (Allan, 1966). Researchers interested in the dynamics of human behaviors utilize fractal analyses in order to identify activity exhibiting long-term sequential dependencies, i.e., behaviors occurring in the past impact present and future behavior. Generally speaking, a time series with higher fractality means that the variance in a behavior at various time scales exhibit similar decay functions, and so there is a power law relationship between behavioral variability and the range of time scales. Fractal analysis may be useful for developmental psychologists interested in the dynamics of limb and vocal behavior because it provides information about whether particular behaviors are impacted by past events or, to the contrary, are occurring randomly. We refer the interested reader to Kello (2013) for a more thorough treatment of the AF analysis applied to a spiking neural network model.

The AF analysis utilizes time series that are point processes, which are time series of events occurring at instantaneous points in time. Examples of discrete time series or point processes are auditory-nerve action potential onsets (Lowen and Teich, 1992) and speech event onsets during conversation (Abney et al., under

review). A typical data series takes the form of a binary spike train: 0 indicates that the event of interest did not occur and 1 indicates the onset of the occurrence of the event of interest. AF analysis takes this time series and first divides it into bins of small time windows. It counts the variability across time bins in the number of events they contain. It then does the same for a slightly larger time bin size, then for an even larger time bin size, and so on. One can then look at the slope, *α*, relating log variability across time bins to log time bin size.

An AF *α* ~ 1.0 can be interpreted similarly to 1/*f* noise (Lowen and Teich, 2005). Previous cognitive scientists have shown that behaviors exhibiting 1/*f* fluctuations reflect cognitive functions that are more flexible and adaptable to changing conditions (Kello et al., 2008), consistent with the notion that 1/*f* fluctuations are signatures of behaviors that are influenced by previous behaviors at increasingly longer temporal scales. Given this, we assume that behaviors with an AF *α* closer to 1.0 (i.e., larger AF *α*'s) will be more influenced by past behaviors, and a behavior resulting in an AF *α* considerably smaller than 1.0 is more random, and less influenced by patterns of behavior from the past. Henceforth, we will use the term *multiscale properties* when discussing the history-dependence of behavior. Behaviors that show more dependence on past behaviors (larger AF *α*'s) have higher multiscale properties because there is a larger correlation between clustering of behavioral events and the timescale of analysis. In contrast, behaviors with less dependence on past behaviors (smaller AF *α*'s) have lower multiscale properties because there is a smaller correlation of clustering of behavioral events across the time scales of analysis and thus, these behaviors are more random.

To our knowledge, no other study utilizes both %DET from RQA and *α* from AF analysis. It is an open question what the relationship between the two measures might be. We will attend to this question in a sub-section of the results section; however, it is of ancillary concern considering our main research goals.

Finally, it is important to note that because of the nature of the data collection procedure—naturalistic day-long recordings—we cannot in this study disentangle the different sources of SWs movements. Possible sources include movements made by SW as part of the behaviors that were also tracked in the milestone diary (e.g., rolling over), other movements made by SW (e.g., fluctuations in posture leading up to rolling over), and movements that were generated by an external entity (e.g., getting picked up by a caregiver). The extent to which each of these types of movement are present in the data, and how much each contributes to the overall activity, RQA, and AF measures, must ultimately be determined through further empirical investigations.

## Case study

The case study reported here is an exploration of the application of nonlinear methods of time series analysis to multimodal infant behavior. We ask the following questions:

1. Do the stabilities and multiscale properties of limb and vocalization behaviors change across infant development?
2. Are there relationships in the stabilities and multiscale properties across these two modalities and effectors?
3. Do the changing dynamics of these modalities and effectors relate to developmental milestones?

### Collection of limb and vocalization data

The present study utilizes a multimodal, longitudinal dataset comprised of daylong recordings of limb movements and vocalizations from one infant, SW. Data collection began when SW was 51-days-old and ended at 305-days-old. During this period, limb movements and vocalizations were recorded on 47 days at a frequency of about once per week. Throughout this time, SW's parents also noted language and motor milestones (e.g., “rolls front to back,” “canonical babbles”) as defined in the existing literature (Adolph et al., 2008; Buder et al., 2013).

Although the movement and vocal recording devices were started/stopped at approximately the same time for each recording session, the recordings cannot be time-locked to a degree of accuracy that would afford detailed analysis of the synchronization between limb movement and vocalization on a moment-to-moment basis. Nevertheless, the analyses implemented herein provide a rich picture of the unfolding structure of infant limb and vocalization development at the level of the day, coinciding with the emergence of important developmental milestones.

#### Recording of limb and vocalization behavior

Recordings of limb movements were made by using Actigraph accelerometers that SW wore around her wrists and ankles (Santos-Lozano et al., 2012). The Actigraphs recorded acceleration of each limb at 100 Hz in three dimensions. Since they do not record tilt, the three-dimensional information is difficult to interpret (i.e., if one wears an Actigraph around the wrist and rotates the wrist by 90° during arm-movements, then acceleration along the vertical axis would be recorded by two different dimensions in the coordinate system of the Actigraph). Hence, we collapsed the three-dimensional information obtained from the Actigraphs to a one-dimensional overall acceleration time series by calculating the magnitude of acceleration for each three-dimensional data point.

Recordings of infant vocalizations were made using the LENA (Language ENvironment Analysis) system, which consists of a small audio recorder that can record for up to 16 hours at a time, custom made clothing with a pocket on the front for the recorder, and a software system for automatically identifying speakers within the recording (Ford et al., 2008; Xu et al., 2009; see Oller et al., 2010; Soderstrom and Wittebolle, 2013; Weisleder and Fernald, 2013; Warlaumont et al., 2014, for examples of other studies that have used the system). The recorder captured SW's voice as well as other sounds in her environment. In the present study, only timings of the onsets of the infant's own vocalizations were considered. Vocalization types included speech-related sounds, such as babbling, singing, and gooing; reflexive sounds, such as cries and laughs; and vegetative sounds, such as burps and grunts. The vocalization onset times were obtained through a custom script that searched for onset times of infant-produced segments within the LENA ITS (Interpreted Time Segments) file (Xu et al., 2008; Warlaumont et al., 2014).

SW's parents began recording when she awoke in the morning and stopped when she was put to bed at night. At times, the audio recordings needed to be paused for privacy reasons. When this occurred, we found the (approximately) corresponding portions of the limb activity recordings and also excluded them from the analysis. In the cases where audio recordings were paused and then started again within the same day, each recording session was considered an individual data point used for subsequent analyses. The recordings lasted ~10 hours on average (ranging from 2.5 to 12.5 h). In total, for each recording session, we had five digital recordings: one each corresponding to the activity in each of the four limbs and one audio recording capturing the child's vocalizations.

#### Developmental milestone data

Qualitative aspects of SW's development were logged by SW's parents throughout the entire data collection period and documented by pictures, diary, and a questionnaire for vocal behavior. The vocal milestones were identified retrospectively based on matching the vocal questionnaire entries with the terms given in Buder et al. (2013), but note that the parents did not refer to the Buder et al. definitions when filling out the questionnaires and instead used their intuitive judgments of the meanings of the terms in the questionnaire. The other motor milestones were identified retrospectively using the pictures and diary entries along with the definitions provided in the Appendix of Adolph et al. (2008). The onset of a milestone was determined as the observation of the first voluntary occurrence of the behavior. More information about the milestones, their definitions, and the vocal behavior questionnaire, is provided in the Appendix.

Unfortunately, it was not possible to identify every single milestone from Adolph et al. and Buder et al. based on these notes, but the parents identified as many as they could. The motor milestones that were identified were “rolls back to front,” “torso raised (propped on arms),” “torso raised (one arm free),” “sits (propped on hands),” “sits (hands free),” “sitting to prone,” “turns 180° prone,” “crawls on belly,” and “prone to sitting.” The vocal milestones were “grunts,” “growls,” “coos,” “laughs,” “vowel-like sounds,” “squeals,” “yells,” “canonical babbling,” and “whispers.”

#### Analyses

It is important to note that there are two levels of measurement being reported in this case study. The first is the level of each recording day. The time series at this measurement level was used to estimate the nonlinear measures computed using RQA and AF, as well as the average acceleration for limb movements and average vocal volubility. In other words, we computed three variables for each day's limb and vocalization activity: (1) the average level of activity (average acceleration for each day recorded and average rate of vocalization for each day), (2) %DET (average deterministic structure in limb accelerations and vocalization for each day), and (3) AF (average strength of multiscale properties for each day).

Once the dependent measures were computed, a longitudinal series of these dependent measures, with approximately one point per week over the course of the data collection period, was constructed. Each of the dependent measures was then correlated with the age (in days) of the infant to assess the individual developmental trends in limb movements and vocalizations over the 254-day period. At this second measurement level, we also used change point analysis in order to detect significant increases and decreases in the dependent measures across SW's development. It was also at this level that we looked at correlations between effectors and vocalizations.

***Overall activity level***. For the limb data, accelerations were averaged per session to obtain overall activity levels. For the vocalization data, overall activity level (i.e., volubility) was operationalized as the number of vocalizations divided by the total length of the recording session. Inter-event-times greater than 15 min were discarded under the assumption that if no vocalization or movement was recorded for 15 consecutive minutes, the infant was likely asleep; this was done for all RQA and AF analyses.

***Recurrence quantification analysis***. For RQA, each recording was converted into a time series of interval durations. For both modalities, interval duration consisted of the duration between each onset of a behavior. For acceleration, the raw, unsmoothed time series were used and the threshold of limb behavior was set to accelerations exceeding 0.05 g. For the vocal modality, the LENA system's labels of onsets of child vocalization segments were used. The parameters (delay and dimension) were calculated for each data set of arm and limb acceleration intervals, and the average values were then used for all data sets to conduct the analysis, resulting in delay = 1, and dimension = 5. Also, all data sets were analyzed with the same radius parameter, which was chosen so that the average %REC across all data sets was 5%, using Euclidean normalization of the phase-space.

***Allan factor analysis***. For the AF analysis, each recording was converted into a binary time series of behavior onsets. Each onset of behavior was marked as a “1” and all other points in time when there were no onsets of behavior were marked as “0.” Time windows varied as a power of 2, *T* = 2*^t^* where *t* ranged from 4 to 12, and therefore ranged from (approximately) 16 s to 68 min. As for the RQA, the threshold of limb onsets was set to 0.05 g and vocalization onsets were given by the LENA speaker segmentation software.

***Change point analysis***. In order to determine whether a change occurred in time series data, we used two change point analytic methods, Taylor's method and MSE. We used both methods to verify that the same changes can be detected using multiple methods, and therefore are reliable and more likely to be real. A large portion of research in developmental psychology revolves around identifying changes in behavior over time (Adolph et al., 2008). Change point analysis provides one analytical method for determining such significant changes in a data series (Buracchio et al., 2010; Kass-Hout et al., 2012). Please see the Appendix for a technical supplement regarding the two change point analytic methods.

### Results

#### Changes in limb and vocal activity patterns over time

Our first question regards the dynamics of vocalization and limb behavior and the changes of these properties over time. Pearson correlations provided a course-grained perspective of the overall developmental trend during SW's first year of life (see Table 1). Variables were standardized for all correlational analyses. For these correlations, a reliable positive correlation represents an increase in a measure and a reliable negative correlation represents a decrease.

**Table 1:** **Limb and vocalization compared to age, Pearson correlation coefficients**.

	**Left leg**	**Right leg**	**Left arm**	**Right arm**	**Vocalizations**
Overall activity	0.462^***^	0.313^*^	0.579^***^	0.615^***^	−0.104
%DET	0.309^*^	0.368^*^	−0.386^**^	−0.356^**^	−0.679^***^
AF	0.267	0.206	0.337^*^	0.391^**^	−0.331^*^

* *p ≤ 0.05*,

** *p ≤ 0.01*,

*** *p ≤ 0.001*.

***Overall activity***. Mean limb acceleration for each of the four effectors exhibited positive correlations with age, suggesting a general pattern of increased movement as SW got older. We did not observe a significant change in vocalization activity. This may be because all types of vocalizations were included in the analysis, including cries and vegetative sounds as well as speech-related vocalizations.

***Change in %DET***. %DET of leg activity reliably increased with time, whereas %DET of arm activity decreased over time. %DET of vocalization activity decreased with time. This suggests that there were increasingly stable patterns in leg activity and decreasingly stable patterns in arm and vocalization activity as SW got older.

***Change in Allan factor estimate***. Recall that a reliably positive increase in AF estimates for arm activity suggests behavior that is correlated across temporal scales and is more dependent on previous behaviors. AF estimates for vocalizations showed a negative trend with time suggesting that the patterns of vocalizations showed less influence from previous behaviors, that is, increasing independence across the period studied (from about 2 to about 10 months of age). AFs increased over time for the arm movements, suggesting decreasing independence. It is worth noting that AF and %DET had different patterns of change over time across the various effectors and modalities.

#### Relationship across modalities and effectors

Our next empirical question regarded the relationships between the modalities and effectors. For example, do the stabilities (%DET) of various limb effectors correlate with each other? Does the degree of stability of vocalization behavior relate to the degree of stability of limb motion? We compared each modality and effector separately across the dependent measures (see Table 2).

**Table 2:** **Correlation coefficients between effectors and modalities**.

	**Vocalizations**	**Left leg**	**Right leg**	**Left arm**	**Right arm**
**LEVEL OF ACTIVITY**					
Vocalizations		0.129	0.223	0.114	0.083
Left leg			0.867^***^	0.626^***^	0.613^***^
Right leg				0.597^***^	0.558^***^
Left arm					0.820^***^
Right arm					
**%DET**					
Vocalizations		−0.292^*^	−0.303^*^	0.282	0.374^**^
Left leg			0.634^***^	0.034	0.064
Right leg				−0.333^*^	−0.167
Left arm					0.708^***^
Right arm					
**AF**					
Vocalizations		−0.008	0.046	−0.058	0.006
Left leg			0.284^*^	0.324^*^	0.331^*^
Right leg				0.905^***^	0.898^***^
Left arm					0.951^***^
Right arm					

* *p ≤ 0.05*,

** *p ≤ 0.01*,

*** *p ≤ 0.001*.

***Acceleration and modality/effector comparisons***. There were no reliable correlations between the degree of volubility of vocalizations and the average accelerations of any of the limbs. Although there were no cross-modal effects of level of activity, there was a markedly strong relationship between the limb effectors. All of the limbs' behaviors were strongly correlated with each other.

***%DET and modality/effector comparisons***. There was a reliable relationship between the vocalizations and limb movements regarding the degree of stability, operationalized as %DET. However, the direction of the relationships depended on the individual limbs. There was a reliably negative relationship between vocalizations and both feet and a reliably positive relationship between vocalizations and the right arm. In other words, more stable patterns of vocalizations were accompanied by more stable patterns in the right arm.

Throughout development, the left and right limbs' %DETs correlated positively within the legs and the arms, exclusively. These results provide evidence for a functional relationship across the left and right effectors for the upper and lower extremities.

***Allan factor and modality/effector comparisons***. Vocalization AF slopes did not correlate with any of the limb effectors' AF slopes. Thus, the multiscale properties of vocalizations did not match that of the limbs. However, there was a reliably positive relationship between all of the limb effectors.

#### Change point analyses

To identify significant changes in the dependent measure across time, we noted change points that were found in both the Taylor change point analysis and the MSE change point analysis. Convergence was considered to occur when either the main or the secondary Taylor change point matched the MSE change point (see Table 3).

**Table 3:** **Change point results for each modality, effector, and dependent measure**.

	**Taylor change point age**	**MSE change point age**	**Secondary Taylor age**
**LEVEL OF ACTIVITY**			
Vocalizations	**157**	**157**	87
Left leg	**108**	**108**	
Right leg	**108**	**108**	122
Left arm	**108**	**108**	
Right arm	**108**	**108**	
**%DET**			
Vocalizations	**150**	**150**	
Left leg	108	**66**	**66**
Right leg	143	**101**	**101**
Left arm	200	291	
Right arm	242	248	
**AF**			
Vocalizations	129	**157**	**157**
Left leg	**297**	**297**	122
Right leg	**248**	**248**	72
Left arm	200	**248**	**248**
Right arm	200	**248**	**248**

*Bold text indicates convergence of two change point methods*.

Change point analysis revealed significant changes in the acceleration data, and the most robust change occurred at the same time in all four limbs, at 108 days of age.

For the measures of vocalization (%DET, AF, and Volubility), convergence of the two change point methods suggested that the point of greatest change was in the range of 150 to 157 days of age.

**Figure 2:**
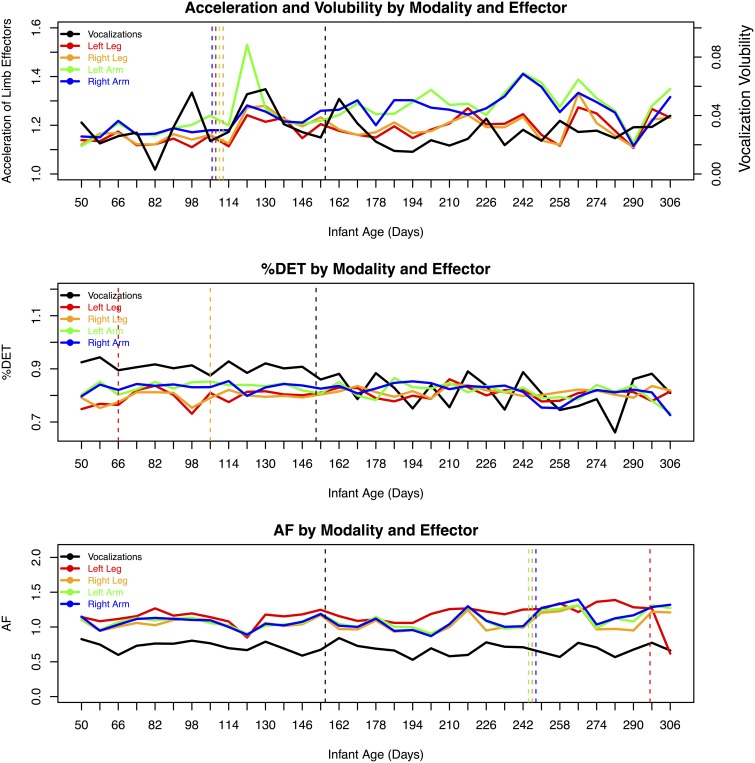
**Overview of the dependent measures as a function of age, modality, and effector**. Change point convergence between both methods are indicated by dashed vertical lines.

Notably, for the limbs, change points in the %DET measures for the legs preceded change points in the Allan factor measures. This might suggest that a change in the repetition of limb patterns precedes changes in the overall multiscale activity distributions of the effectors. Figure 2 provides an overview of the longitudinal data series of dependent measures and indicates days where significant changes occurred.

#### Developmental milestones

**Figure 3:**
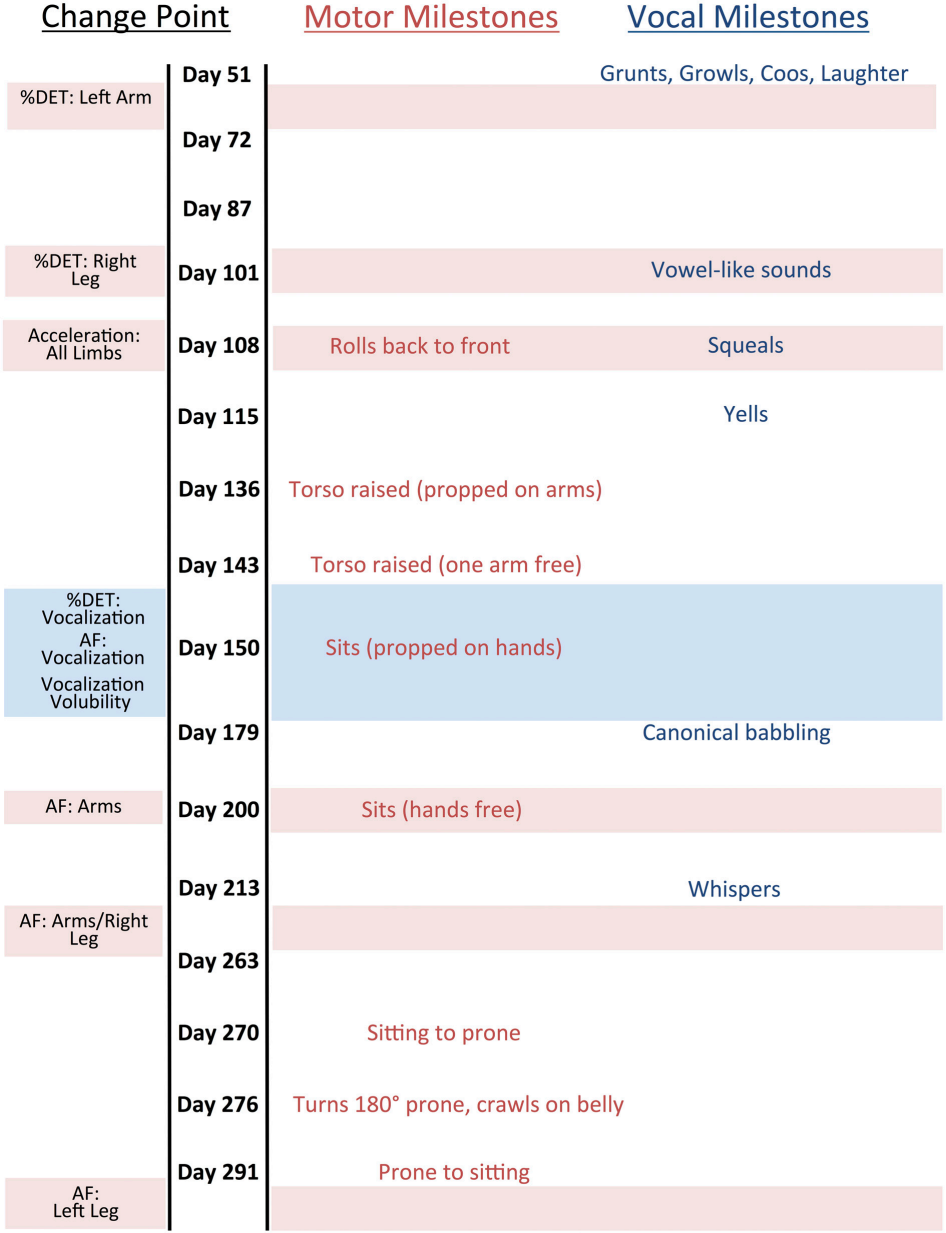
**Change Point Results with Language and Motor Milestones**. Horizontal bars correspond with particular change points. Age does not increase linearly but rather as a function of recording session.

Figure 3 shows change point days relative to motor and language milestones. Recall that SW's parents documented the dates of achievement of a variety of apparent motor and language milestones that occurred during the recording period. A qualitative analysis of these data suggests that some converging change point dates coincide with or shortly precede important milestones.

Convergence of change points for all vocalization measures (%DET, AF, Volubility) directly preceded the language milestone “canonical babbling.” As discussed in the introduction, the onset of canonical babbling is considered evidence of a growing capacity for speech and provides the basis for more complex speech-related vocalizations (Eilers et al., 1993; Oller, 2000). This suggests that there may be a number of changes in an infant's vocal communication system that are leading up to the onset of canonical babbling, which would further highlight the significance of this motor milestone.

## Discussion

This case study explored the nonlinear dynamics of limb and vocalization behaviors. The goals were to investigate: (1) the changing stabilities and multiscale properties of limb and vocalization behaviors across the first year, (2) the relationships across the two modalities, and (3) the relationship between the changing limb and vocal dynamics and parent-reported developmental milestones.

### Functional dissociation and selectivity of limb behaviors

There were a number of intriguing findings warranting further study with a larger sample of infants. We saw—unsurprisingly—that limb activity increases throughout the first year in all limbs. The nonlinear measures of %DET and AF enrich this picture: %DET indicates that the overall movement activity of the legs becomes more repetitive and stable with age, while the inverse pattern is observed in the arms. Furthermore, AF indicates that movements of both arms become increasingly dependent on previous behaviors, that is, they show increasingly correlated clustering across timescales, while this is only marginally the case for leg movements. This might reflect the development of a functional dissociation between arms and legs (Kanemaru et al., 2012), where leg activity becomes increasingly confined to a smaller set of movement patterns, while coordination and diversification of arm movements increases (Wallot et al., under review).

Across limbs we observed correlations between left-right effectors for the upper and lower extremities, respectively. This was predominantly salient for the %DET measure of recurrent temporal patterns of activity. This is another good example of the departure between linear and nonlinear metrics of movement behaviors. Kanemaru et al. (2012) observed no differences in velocity or amplitude over time, but importantly, increased correlations between right and left arms and right and left legs in velocity and position. They suggested this pattern might facilitate goal-directed behaviors like reaching or grasping. Our %DET results suggest similar evidence for dissociation and flexibility. With the current dataset, it is not possible to identify specific goal-directed actions (e.g., reaching or grasping) that this might facilitate, though it is possible that the increase in dissociation and flexibility are correlated with increasingly skillful behaviors. The increased coordination between the two legs and the increased stability of the legs' dynamics might be indicative of a facilitative role for motor achievements like unsupported sitting. Previous work has shown that the stability of COP in postural control was indicative of greater sitting ability (Harbourne and Stergiou, 2003). The decreased stability in the coordinative patterns of the arms might suggest an exploration of degrees of freedom, which facilitate transitions—phase shifts—to producing new, more skilled behaviors (Thelen et al., 1987, 1993). We hypothesize that this reduction in stability of arm movements might also relate to the achievement of the language milestone canonical babbling (Eilers et al., 1993; Ejiri and Masataka, 2001; Iverson and Fagan, 2004), which will be discussed later.

It should be noted that it is possible that some of the recorded accelerations were due to circumstances not controlled by SW, such as being moved by an adult. The recorded accelerations also included those that happened during the execution of the milestone behaviors and those that were separate from those specific behaviors. The data should therefore be thought of as reflecting all SW's limb movement experiences. For some purposes, all sources of information may be viewed as potentially significant; for other purposes, it will be important to filter out some types of movement sources. Regardless, this lack of information poses a challenge for interpretation of our results. Future work would benefit from the development of automated filtering procedures to parcel out the different sources of infant movement.

### Reduction in stable vocalization patterns

For vocalizations, we did not observe a correlation between volubility (i.e., vocalization rate) and age. Nevertheless, there were still marked vocalization patterns observed for the analyses introduced here, indicating that nonlinear approaches can at least in some cases provide more sensitive measurements of changing vocalization patterns. The nonlinear measures revealed developmental trends: we observed a trend toward lower stability and less multiscale dependency in utterance timings. Together, these might index a growing capability on the part of SW to use vocalizations in communicative interaction with other individuals (Jaffe et al., 2001; Ramsdell et al., 2012). The decrease in %DET suggests a diversification of utterance timing patterns over time (Fusaroli and Tylén, under review), while the decrease observed in AF indicates a decline in endogenous vocalization-fluctuations over time toward a more locally determined pattern of vocalization timing (e.g., Kuznetsov and Wallot, 2011). We hypothesize that these patterns may relate to SW's vocalizations becoming more context-sensitive.

Although there was a reduction of AF over time, it is important to note that any vocalization structure at all, i.e., AF>0, indicates a non-Poisson process, and therefore non-random vocalizations. Many previous theorists (Mowrer, 1952; Jakobson, 1962; Lenneberg, 1969) have argued that babbling is a random motor act. Of course, it is difficult to find any biological organism that does not produce structured, non-random signals, whether behavioral, physical, or cognitive (Kelso, 1995). Previous research has indicated that there is hierarchical structure in pre-canonical protophone production even during the first year of life. Specifically, hierarchical phrasing, identified by adult judges, has been observed as early as 3-months-of-age, and this ability has been shown to be attenuated for infants diagnosed with Downs syndrome (Lynch et al., 1995). The results from the AF analysis of SW's vocalizations indicates that there were hierarchical patterning of her vocalizations as early as 2 months and throughout the rest of her first year, at larger timescales, ranging from (approximately) 16 s to 68 min. In the future it would be interesting to test the idea that the hierarchical organization detected here is reflective of proto-conversational (see also Jaffe et al., 2001) and proto-discourse capabilities.

### Limb-vocal coordination and canonical babbling

We also observed a co-evolution of the regularity and stability of limb movements and vocalizations. First of all, when leg activity became more stable, arm and vocalization activity became less stable, and thus more diverse. In other words, arm movements and vocalizations “diversified” together. This complements previous studies documenting relationships between canonical babbling onset and rhythmic limb movement (Eilers et al., 1993; Cobo-Lewis et al., 1996; Ejiri and Masataka, 2001; Iverson and Fagan, 2004), suggesting that the extent of coordination across different infant behaviors may be greater than previously thought. Previous experiments employed more intricate designs that either coded the onset of canonical babbling from video recordings (Ejiri and Masataka, 2001) or created a cross-sectional sample of infant groups that differed as a function of babbling experience (Iverson and Fagan, 2004). These studies also coded particular properties of limb actions and vocalizations which afforded an in depth investigation into the functional relationships between different specific behavior types. In contrast, we studied the longitudinal pattern of behavior at a more macro level over the first year of SW's life. We relied on parental report of canonical babbling (Oller et al., 2001) and we also have not (yet) coded for particular limb action and vocalization properties.

Thus, while it has its limitations, our study complements previous research on limb-vocal coordination and language development, extending that work in new directions. Before we discuss these additional insights from the current work, it is again important to note that the lack of information regarding the differentiation between endogenous and exogenous limb movements and movements independent of milestones makes our interpretations tentative without subsequent data filtering.

First, we found that the largest changes in all three measures of vocalization patterns changed directly preceding canonical babbling. This finding provides further support for the significance of canonical babbling as an early speech development milestone and suggests that it may be associated with broader changes in infant vocalization patterns at a range of longer timescales.

Second, we found that the right arm and vocalizations both decreased in stability with age. This observation supports the notion that these effectors are coordinated and that they are both increasingly flexible over the first year, suggesting a coordinated exploration phase of limb-vocal development. Ejiri and Masataka (2001) and Iverson and Fagan (2004) used a variety of measures that quantified the rate of co-occurrence of limb movements and vocalizations to determine trajectories that precede and coincide with canonical babbling. For example, Ejiri and Masataka (2001) found that a high frequency of co-occurrences preceded the onset of canonical babbling. Our results suggest that particular properties of limb movements and vocalizations, namely, a nonlinear index of stability, are indicative of canonical babbling, too. Future work should consider estimating nonlinear measures of limb and vocal behaviors in a larger sample of children, pre- and post-canonical babbling onset, to confirm that these measures are predictive of the onset of canonical babbling and if so, determine which are most predictive.

Third, it is intriguing to consider the co-development of rhythmic limb behavior and vocalizations as two interdependent components of a larger communicative system (see Yale et al., 2003; Parladé and Iverson, 2011). In particular, future work might investigate if changes in stable motor patterns—whether from the limbs or vocalization—point to the emergence of various communicative gestures (Iverson et al., 2008).

Finally, our results point to a laterality effect found in adult speakers and infant samples (Iverson and Fagan, 2004) using coexpressive gestures (Kimura, 1973; McNeill, 2000). Adult coexpressive gestures are typically observed to be unimanual and produced with the right hand (Kimura, 1973). Iverson and Thelen (1999) proposed a developmental model wherein infant motor-vocal coordination serves as the origin of gesture-speech coordination in older children and adults. Evidence for a laterality effect was found by Iverson and Fagan (2004), showing that infants were more likely to coordinate vocalizations with unimanual (relative to bimanual) movements, and a higher proportion of these unimanual movements were observed to be right-handed. We found a relationship between the degree of stability of right-arm movements and vocalizations, providing further support for the idea that the right arm and vocalizations are coordinated from an early age. Again, future research with a larger sample is warranted.

### Nested actions in unsupported sitting and canonical babbling

One last observation is the temporal proximity between the postural milestones of “Sits (propped on hands)” and “Sits (hands free)” with change point results for the vocalization properties and also the canonical babbling milestone. Yingling (1981) showed that unsupported sitting facilitates: (1) greater control over utterance production, (2) increase in consonant-vowel units, and (3) increase syllable production per breath (see also Boliek et al., 1996). Taken together, these findings suggest a nested structure of limb and vocal actions (Reed, 1982, 1996; Gibson and Pick, 2000). Nesting of action capabilities (see Nickel et al., 2013) have profound consequences for future action, or “cascading effects” (Turvey, 1992; Koterba et al., 2014). Transitions from crawling to walking afford enriched visual information (Kretch et al., 2014), increase social interactions (Biringen et al., 1995), and even accelerate language development (Walle and Campos, 2013). Unsupported sitting might also serve as an important precipice for subsequent, cascading actions in typical-developing and at-risk populations (Nickel et al., 2013).

## Conclusion

This study demonstrates that approaching the infant as a complex system with the tools from nonlinear dynamics allows for novel characterizations of infant behavioral development. We have shown that for infant SW particular nonlinear measures descriptive of the properties of a developing complex system (a) changed as she grew older, (b) exhibited differences across modalities and effectors, and (c) preceded important developmental milestones. While the current study is inherently limited by its single-case nature, the interesting patterns of dynamics observed for the individual child studied in depth here demand future work testing whether any of these patterns hold more generally across children.

### Conflict of interest statement

The authors declare that the research was conducted in the absence of any commercial or financial relationships that could be construed as a potential conflict of interest.

## Footnotes

^1^Co-authors Wallot and Haussman.
