# Supplementary material for: Using nonlinear methods to quantify changes in infant limb movements and vocalizations
Source: Front Psychol. 2014 Aug 12;5:771. doi: 10.3389/fpsyg.2014.00771 (PMC4130108; doi:10.3389/fpsyg.2014.00771)
Supplement: Supplementary file 1 [file DataSheet1.PDF]

Appendix

Taylor's method

Taylor's method for change point computation is based on the mean-shift model and assumes that data points are independent of each other and identically distributed. The method uses cumulative sum (CUSUM) charts to determine if and when a change has occurred, and what the direction of that change is (Taylor, 2012; Mustonen et al., 2012). It is a valuable method to use independently or in a complementary fashion with other methods because it can detect subtle changes that can be missed with other methods, it is robust to outliers, and it is easy to implement (Kass-Hout et al., 2012).

The procedure requires calculation of a cumulative sum (S_i) for each data point in the series, resulting in a series of sums from S_0 to S_n, where $S_0 = 0$. The sum for each point is calculated by adding S_{i-1} to the difference between the data at that point and the mean of all the data (Mustonen et al., 2012):

$$S_i = S_{i-1} + (x_i - \bar{x}).$$

A positive slope of S_i indicates that the values involved are above the mean and a negative slope indicates that those values are below the mean. A shift in slope direction indicates a shift in the mean, allowing us to infer that a significant developmental change has occurred at that point.

MSE CP

Another method for determining change-point is the mean square error (hereafter, MSE CP). The MSE CP involves splitting the data series into two halves (1 to m, and m+1 to n), calculating the mean for each half, and then evaluating how well the data in each half relates to the mean for that half of the data series. That is, m is the last point preceding where the change might have occurred, and m+1 is the point just after where the change might have occurred. The value of m that minimizes MSE(m), the error between the data and the two means, is the best estimate of the last point before the change occurred (Taylor, 2012):

$$MSE(m) = \sum_{i=1}^{m} (x_i - \bar{x}_1)^2 + \sum_{i=m+1}^{n} (x_i - \bar{x}_2)^2$$

$$where\ \bar{x}_1 = \frac{\sum_{i=1}^{m} x_i}{m}\ and\ \bar{x}_2 = \frac{\sum_{i=m+1}^{n} x_i}{n-m}$$

where n is the total number of points in the series.

Motor milestone skills and their definitions (adapted from Adolph et al., 2008)

Skill	Operational definition
Rolls back to front	Shifts from lying supine to lying prone.
Torso raised (propped on arms)	Pushes head and chest off floor by propping on forearms or hands while lying prone.
Torso raised (1 arm free)	Pushes head and chest off floor by propping on 1 arm and using the other hand or arm to reach or manipulate objects.
Sits (propped on hands)	Sits on floor for more than 30 s, with legs outstretched, using hands for support.
Sits (hands free)	Sits on floor for more than 30 s, with legs outstretched, without using hands for support.
Sitting to prone	Shifts from sitting position with legs outstretched to prone position.
Turns 180° prone	Pivots in place more than 180° while on belly or hands and knees.
Crawls on belly (less than 3 m)	Crawls forward less than 3 m, before stopping, with belly resting on floor for duration of each crawling cycle.
Prone to sitting	Shifts from prone or crawling position into sitting position with legs outstretched.

Vocal milestones and their definitions (adapted from Buder et al., 2013)

Skill	Operational definition
Grunts	Vegetative sound, not specialized as a communicative signal, produced reflexively (as an accompaniment of movement or physical straining).
Growls	Growls as phonation-related protophones have either very low pitch, below that of the infant's habitual voice, or they can be within the habitual pitch range but have a harsh or noisy quality.
Goos	Goos are vocalizations with very primitive articulation where a tongue closure articulation in the back of the oral cavity is superimposed upon phonation, usually normal phonation. While goos do include articulation, the product usually does not yield clear syllabification, but rather seems only to hint at the infant's emerging potential for syllabification by articulation.
Laughs	Laughs are species-specific vocalizations that are strongly associated with a fixed set of social and emotional contexts. When they are produced in an apparently reflexive way they are considered to be similar to sounds of other mammals and thus to be less speech-related than the protophones.
Vowel-like sounds	The vocants, both quasivowels and full vowels, are also defined by the auditory impression of normal speech-like phonation and pitch (in contrast with the phonation and pitch characteristics of squeals and growls). The phonatory and pitch characteristics of vocants must be within the infant's "habitual" range.
Squeals	Squeals have extreme high pitch, beyond the range of the infant's habitual voice.
Yells	Yells are notably loud sounds, and usually have a pressed quality, but the defining

	quality with yells is that they are produced with amplitude that is beyond the habitual range of the producer's voice.
Canonical babbling	Sounds with syllable-like patterns generated by supraglottal articulation. Canonical babble has speech-like speed of timing of the transitions between consonant-like and vowel-like portions of the syllable.
Whispers	Whispers are quiet and not voiced, though the air can be heard passing through the laryngeal cavity.

Vocal milestone diary questionnaire:

Your name:
Your baby's name:
Date when this form was filled out:
Dates reported in this form:

1. What kinds of sounds did your baby make during the period since we last spoke?

2. With regard to particular sound types (crying, laughter, vowel-like sounds, squeals, growls, raspberries, yells, whispers, marginal babbles, canonical babbles, others) did your baby produce them during this period?
   How often (occasionally, quite a bit, very often)?

3. How often did the baby vocalize independent of the type of vocalization produced?

4. In what circumstances (in interaction, when alone, when in the same room or nearby) did the baby vocalize?
   How often?

5. Does the baby repeat sounds either imitatively or when alone?
   How often?

6. Does the baby engage in turn taking vocalization with you or others?
   How often?

7. Does the baby call to you for attention?
   How often?

8. Does the baby talk to toys and produce sounds in solitary play that seem to be directed to objects?
   How often?

9. Does the baby produce playful vocalizations in social interaction?
   How often?

10. Diary Questionnaire for children who are in the canonical stage but not yet up to 50 words in production as indicated by MCDI:
    Please list the syllable or syllable sequences that your child produces regularly.

11. Any additional comments or feedback?

Thank you for participating!

References

Adolph, K. E., Robinson, S. R., Young, J. W., and Gill-Alvarez, F. (2008). What is the shape of developmental change? *Psychol. Rev.* 115, 527–543. doi: 10.1037/0033-295X.115.3.527

Buder, E. H., Warlaumont, A. S., and Oller, D. K. (2013). “An acoustic phonetic catalog of prespeech vocalizations from a developmental perspective,” in *Comprehensive Perspectives on Child Speech Development and Disorders: Pathways From Linguistic Theory to Clinical Practice*, eds B. Peter and A. N. MacLeod (New York, NY: Nova Science Publishers), 103–134.

Kass-Hout, T. A., Xu, Z., McMurray, P., Park, S., Buckeridge, D. L., Brownstein, J. S., et al. (2012). Application of change point analysis to daily influenza-like illness emergency department visits. *J. Am. Med. Inform. Assn.* 19, 1075–1081. doi: 10.1136/amiajnl-2011-000793

Mustonen, A., Lempiainen, T., Aspelund, M., Hellstedt, P., Ikonen, K., Itamies, J., et al. (2012). Application of change-point analysis to determine winter sleep patterns of the raccoon dog (Nyctereutes procyonoides) from body temperature recordings and a multi-faceted dietary and behavioral study of wintering. *BMC Ecol.* 12:27. doi: 10.1186/1472-6785-12-27.

Taylor, W. A. (2012). *Change-Point Analysis: A Powerful New Tool for Detecting Changes.* Available online at: http://www.variation.com/cpa/tech/changepoint.html.
